# Format Changes Improve Learner Satisfaction in Local Neonatal Resuscitation Program Skills Education

**DOI:** 10.7759/cureus.30632

**Published:** 2022-10-24

**Authors:** Leigh A McGlinn, Nathan C Sundgren

**Affiliations:** 1 Neonatology, Texas Children's Hospital, Houston, USA

**Keywords:** situated cognition, simulation, education, technical skills, neonatal resuscitation program

## Abstract

Background: The Neonatal Resuscitation Program (NRP) is a national education program that prepares learners to resuscitate newborn babies. Our local Texas Children’s Hospital (TCH) NRP educational class incorporates skills training and high-fidelity simulations of neonatal resuscitation scenarios. The skills training had four elements we believed were not ideal. 1. Learners went to skills stations in groups, but different teams were formed for performing the scenario simulations in the latter half of the class. 2. The skills stations were visited in random order and not necessarily the actual order in the skills would be performed in a clinical situation. 3. Educational content presented at the skills station was highly variable depending on the instructor. 4. Emphasis was on instructors teaching content over learners practicing and demonstrating skills. In March 2019 we restructured the skills education portion completely to address all four of these problems.

Objectives: To determine how changes to the design of our skills education affected learner satisfaction with the NRP course.

Methods: Using the principles of situated cognition, the skills education was restructured by leadership planning and consensus. We made four changes. 1. Teams were assigned from the beginning of class, and these teams practiced all skills and performed the simulations together. 2. Every team went through the skills stations in the order they would perform them in an actual resuscitation. 3. Videos were made to teach the “gold standard” information needed for the skills stations. 4. Instructors were asked to think of themselves as coaches and not teachers, letting the videos do the education, and they focused on improving skill performance. A survey was designed and disseminated by e-mail to all learners of the first 13 classes taught using the new educational format (n=196). The survey asked learners to compare their experience of the new format compared to their memory of the previous format.

Results: A total of 163 learners responded and completely finished the survey (83% response rate). Of the 163 respondents, 109 learners (67%) had taken the course in the past at TCH and filled out the survey questions comparing the new format to the past format. Out of the total, 93% of learners (n=101) judged the new format to be “better” or “much better.” The percentage of learners that attributed the improvement to the changes we made were: 70% for team assignments from the beginning (n=76), 74% for skills performed in order (n=81), 68% for video-based education (n=74), and 76% for instructors as coaches (n=83). Learners rated the videos used for education very highly.

Conclusions: The reformat of our skills education has improved our learners’ experience during NRP training. The highest-rated change was asking our instructors to operate as coaches rather than teachers.

## Introduction

The Neonatal Resuscitation Program (NRP) is an evidenced-based training curriculum that was created in 1987 to ensure neonatal teams effectively stabilize and resuscitate neonates in the delivery room [1]. The course has changed from a more traditional lecture-based environment to a simulation-based program with hands-on learning over the course of new editions. While NRP provides content goals for learners to know and demonstrate, it does not give specifics on how to teach these skills. The cognitive content is primarily taught in an online portion and is assessed by online multiple choice questions before arrival to the in-person portion of the education program. The hands-on sessions, therefore, are designed to demonstrate technical skill performance (such as positive pressure ventilation, chest compressions, etc.) and emphasize critical leadership, teamwork, and communication skills.

As we took over program leadership for NRP education at our institution, we critically observed the class and contemplated what changes can best enhance our learners’ in-person experience during the technical skills education and formative performance assessment.

## Materials and methods

Setting and context

Texas Children’s Hospital's NRP training program is responsible for over 900 learners annually. The NRP training at our institution incorporated high-fidelity simulation for scenario-based simulations in 2009. However, task trainers were still used in skills education, and the format of the class did not substantially change from that time other than content was updated for each NRP edition. Each class session was four hours and time was divided into individual skills training followed by team-based neonatal stabilization and resuscitation scenarios. Learners were taught in multidisciplinary groups including, doctors, advanced practice providers, nurses, and respiratory therapists. Disciplines included primarily pediatric residents, neonatology, obstetric residents, and newborn medicine. At the time of our study (2019), classes were held 12 to 14 days a year. There were two class sessions lasting four hours each day. This resulted in a total of 24 to 28 sessions a year. Each session held a maximum of 24 individual learners with a typical 3 to 1 learner-to-instructor ratio. Learners came prepared by having taken online course content from NRP through an online learning platform.

Previously, the skills training portion consisted of four skills stations. 1. Initial steps: this station primarily consisted of knowing the four pre-birth questions and drying, warming, and stimulating the patient (mannequin). 2. Bag-mask Positive Pressure Ventilation (PPV) and chest compressions (CC). 3. Intubation: Learners performed or prepared for endotracheal intubation. 4. Emergency umbilical venous catheter (UVC) placement: Learners prepared for or practiced insertion of UVC and drawing up code doses of medications. At the beginning of skills station training, learners self-formed groups of three to four learners. Groups then went in random order to each skill station as a station was found open and available for practice. At each station, NRP instructors taught the basic knowledge content for that station and then the learners performed the skill on low-fidelity or task trainer mannequins. The instructor then “checked off” the learner for that station and the learner group moved to another station until all four stations were completed. Each station took variable time to complete, usually 10 to 15 minutes. The time allotted for all the skill stations to be completed was one hour.

Problem

When we took over as the new leader of the program, we critically assessed our current program. The course was routinely rated very high on the Likert scale used by our simulation center’s post-course survey, but we assessed that there was an opportunity to improve the skills education portion of the class. Technical skills education was fragmented and not situated in realistic scenarios for our learners. Situated cognition, as part of situativity theory, posits that learning is situated in experience and that experience includes the learners, the culture, and the physical environment where the learning occurs [2]. The fragmented approach to the technical skills did not allow the learning to occur in a context that would be similar to neonatal resuscitation in real practice.

We identified four main problems with the course format and skills education. (1) Groups self-formed in the beginning. The groups tended to form along disciplinary lines, and they were never the team they were going to be a part of for the scenario-based simulations. The residents often formed one group separate from any nurses or respiratory therapists. This made stations, such as intubation, longer for this group where everyone in the group had to perform all steps compared to groups of all nurses that performed only the preparation for intubation. From a situated cognition perspective, the learners missed out on learning interactions with other disciplines that they would be performing simulated resuscitations with and not in the context of a multidisciplinary team as in real resuscitation situations [2]. (2) The random order sequence of the skills was not ideal. The random order did not reinforce the correct sequence that each skill needed to be performed in the NRP algorithm. (3) Content taught by the instructors at each station was variable and not always perfectly aligned with the NRP textbook. We noted striking variety in what was said by individual instructors during our assessment phase. (4) Instructors often spent a large amount of time reviewing the information the learners should have learned from the online content. This frequently left groups scrambling at the end to practice the skill a few short times rather than spending most of their time practicing the skill.

Intervention

**Figure 1:**
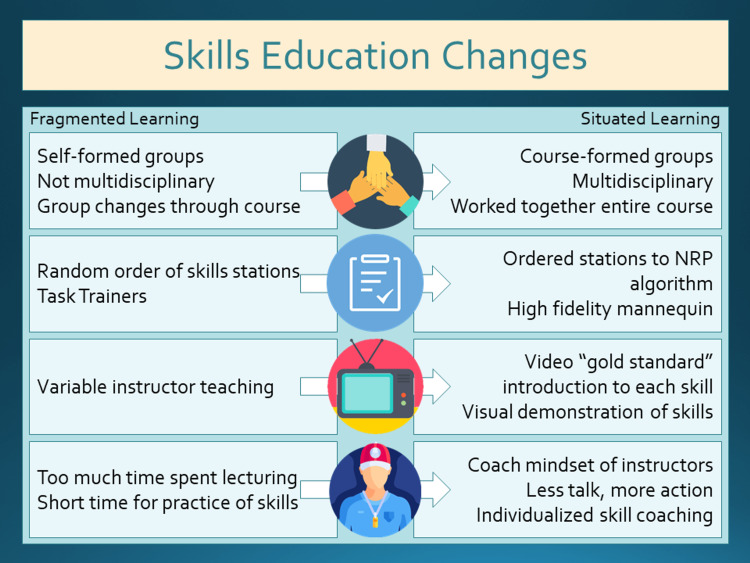
Changes to Skills Education NRP: Neonatal resuscitation program

Our leadership and simulation team met and came up with changes to address each problem identified (Figure 1).

1. Multidisciplinary teams were assigned from the beginning of class. This ensured that the teams worked together throughout the course before working together in the scenario-based simulations, and the different disciplines would not practice in silos. This also had the advantage of aiding learners in understanding different disciplines' capabilities in preparation for the team-based simulations.

2. Every team went through the skills stations in the order they would perform them in an actual resuscitation. The necessary skills were rearranged into three sections instead of four. Learners performed the initial steps and PPV in the first station. Then they performed intubation (alternate airway) and chest compressions for the second station. Lastly, they performed UVC placement and medication administration. Each section of skills was given 20 minutes of time to complete making the total time for skills education the same one hour as before any changes. These changes required each team to have its own high-fidelity mannequin that could accommodate the practice of all the skills and remove the task trainers used in the past.

3. Videos were made to teach the “gold standard” information needed for the skills stations. The use of videos has been demonstrated to be a very useful tool in education [3-5]. Videos can be especially helpful to demonstrate skills, proper sequencing, communication, and teamwork. Utilizing brief videos reduced the cognitive load and improved student memorization and performance when used prior to simulation-based training [6]. Certain aspects of how the videos are made also can contribute to learning. Emphasizing important points through on-screen text or symbols and breaking up important learning content into smaller more specific videos boosts learner retention [7]. Using only the most important points in videos and matching them with on-screen demonstrations has shown learner enhancement [7-9]. Three videos were made to introduce each skill station using these best practices as much as possible. We made the videos to refresh learners’ memories of the key information for each new skill station section. Each video is short at about six minutes long. The text was interspersed with video to emphasize important points (Videos 1-3). Video three was remade from a publicly available video on YouTube on Emergency UVC placement that we considered excellent teaching. Our UVC placement video was created by remaking the video with permission from the video creators (Bobbi Byrne, MD, and Marya Strand, MD)

**Video 1:** Neonatal Resuscitation Initial Steps and Positive Pressure Ventilation

**Video 2:** Neonatal Resuscitation Alternate Airway and Chest Compressions

**Video 3:** Neonatal Resuscitation Emergency UVC and Epinephrine Dosing UVC: Umbilical venous catheter

4. Instructors were asked to function as coaches and not teachers. Instructing is a very direct form of communication and is reliant on the instructor's ability to provide clear and concise direction or instructions [10]. Each instructor brings their own experience, education, bias, and teaching capabilities [2]. This can result in learners not being taught the core objectives, poor use of time, and content being missed or improperly emphasized. Coaching relies on the coach's ability to assess the learner’s technical skill performance while offering corrections to improve performance. This observation and direct feedback are essential to performance improvement [11]. The learners in this model get individual attention based on their learning needs and skill level with specific feedback on their performance of the technical skill. We emphasized to our instructors in pre-brief before each class session that the online NRP content and videos do the education and they focus on improving the performance of the skill. This allowed all the time at the skill station to be utilized as practice and demonstration of the skill. We circulated during each class day between the groups to reinforce the coaching concept to our instructors as we saw the need. In May 2019, we introduced all four changes simultaneously into our in-person curriculum.

Outcome

Our primary goal was to make the class better than before for learners. No previously validated survey existed, that we were aware of, to evaluate our specific changes. Therefore, a survey was designed to assess repeat learners’ impressions of the changes to the class structure (Appendix 1). The survey was distributed by e-mail to all learners in the first eight classes using the new format. First-time learners received the survey, but once they answered that it was their first time taking NRP at our institution, the survey ended for them. The survey asked the learners to compare their experience of the class before to the current structure. They were also asked four questions about the videos made that introduced each skill station. Later, we realized that first-time learners should be able to assess the videos without having had any comparison to previous classes that did not use videos. The survey was changed to allow first-time learners to answer the four questions about the videos before their survey was complete. This survey was distributed to the next five classes. E-mail invitations were sent within a week following the scheduled class date. Non-responders received weekly e-mail reminders two additional times and then daily for one week. All responses were anonymous. The database survey (REDCap; https://www.project-redcap.org/) was configured to track if the learners invited had responded, but the results did not reveal any identifying information.

## Results

A total of 196 learners attended the NRP classes under this study. All learners were sent survey invitations, of which 29 did not respond and four did not complete the survey and were excluded, leaving 163 learner surveys (83% response rate). Of all survey responders, 54 (33%) were first-time learners and 109 (67%) were repeat learners at our NRP program.

Repeat learners (n=101, 93%) overwhelmingly rated the class as better (n=35, 32%) or much better (n=66, 61%). Eight (7%) repeat learners rated the class as similar to before. No one rated the class as worse or much worse. Table 1 shows the percentage of learners that rated each of the four changes we made as a reason the class was better. Multiple answers were allowed, and a high percentage of learners chose all four of the changes we made as better than the previous way skills stations were taught. When answers were only used from the learners that rated the new skills education as better or much better (n=101) all four changes were individually selected as better than the previous by over 70% of these learners. Very few learners felt that none of our changes were better than the previous method (n=7, 6%).

**Table 1:** Learners' responses on which component of change made the class better Our changes were rated as "better" or "much better" by 93% (101 of 109) of our repeat learners taking our survey. This table shows the percentage of repeat learners rating each individual component of our changes as making the class better.

	Repeat Learners (n=109)	Repeat Learners rating class as better or much better (n=101)
Early Team Assignment	76 (69.7%)	75 (74.3%)
Video Education	74 (67.9%)	74 (73.3%)
Skills in Order	81 (74.3%)	79 (78.2%)
Instructors as Coaches	83 (76.1%)	81 (80.2%)
None of these	7 (6.4%)	3 (3.0%)

**Figure 2:**
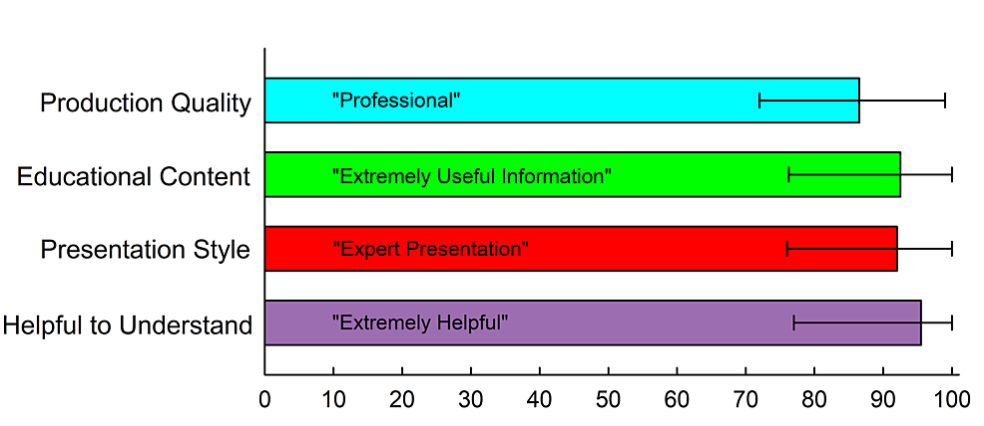
Quality of Videos Used in the Course Learners used a sliding scale to score the videos used in the class. The scores were a sliding scale from 0 to 100 with descriptive verbal anchors at 0, 50, and 100. The quoted text within the bars is the verbal anchor that was closest to the median score results. The box length is the median and the whiskers are the 25th and 75th percentile.

The videos were assessed by the learners in four categories: production quality (how well they were made), educational content (how well the information was taught), the presentation style (how clear and effective the presentation was), and helpfulness (how useful the videos were to help learners understand the content). Learners used an unnumbered slider bar to score each category. Anchor descriptions were given to each end and the middle of each slider as seen in the survey (Appendix 1). The database converted the position of the slider into a number from 0 to 100. Figure 2 shows the results of learners' scoring. A total of 136 learners rated the videos. First-time learners from the first 8 classes (n=27) had surveys that did not allow them to score the videos. Results are shown for first-time learners from the subsequent five classes (n=27) along with the 109 repeat learners. First-time learners’ scores were compared to repeat learners’ scores. There was no statistical difference in median scores given by the first-time learners compared to repeat learners in any of the four categories (p>0.05, Mann-Whitney Rank Sum test). However, repeat learners that ranked the class as similar to previous experience gave much lower scores to the videos compared to repeat learners who ranked the class as better or much better and first-time learners (Table 2).

**Table 2:** Comparing First-Time and Repeat Learners' Scoring of Quality of Videos *denotes a significant difference between repeat learners that rated the class as similar to previous experience compared to repeat learners that rated the class as improved and first-time learners: Educational Content p=0.021, Presentation p=0.016, Helpfulness p=0.035, Kruskal-Wallis One Way ANOVA on Ranks.

	Score, median (interquartile range)
Production Quality	
First-time learners (n=27)	89.0 (72-100)
Repeat learners rating class better (n=101)	87.0 (73-99)
Repeat learners rating class similar (n=8)	58.5 (50-87.5)
Educational Content	
First-time learners	95.0 (80-100)
Repeat learners rating class better	94.0 (76.5-100)
Repeat learners rating class similar	61.0 (50-85.75)*
Presentation	
First-time learners	94.0 (82-100)
Repeat learners rating class better	93.0 (77-100)
Repeat learners rating class similar	53.0 (50-85.5)*
Helpfulness	
First-time learners	100.0 (87-100)
Repeat learners rating class better	95.0 (77-100)
Repeat learners rating class similar	71.5 (50-96.5)*

In addition to our survey, every learner takes a generic post-course survey created by our simulation center before leaving the course for the day. These scores have consistently been high on a five-point Likert scale. Scores were compared from the last seven course days before the skills education changes (n=174 learners) and the first seven course days after the change (n=189 learners). Learners were asked on a five-point Likert scale from “strongly disagree” to “strongly agree” to respond to the statements as to whether the class had improved their clinical knowledge, their technical skills, their communication skills, and whether their instructor made them feel comfortable. There was no statistical difference in the categories (Table 3).

**Table 3:** Simulation Center Survey Results for Course Comparing Before and After Changes Were Implemented High scores were maintained after our changes were implemented. Average score from a five-point Likert scale where 1=strongly disagree and 5=strongly agree. T-test used for statistical comparison. No comparison was significant.

	Before Changes (mean ± std dev, n=174)	After changes (mean ± std dev, n=189)	P Value
This class improved my clinical knowledge	4.78 ± 0.45	4.82 ± 0.43	0.35
This class improved my technical skills	4.73 ± 0.56	4.79 ± 0.41	0.25
This class improved my communication skills	4.79 ± 0.48	4.70 ± 0.49	0.10
My instructor made me feel comfortable	4.82 ± 0.43	4.89 ± 0.33	0.12

## Discussion

Our technical skills education was fragmented and not situated in realistic order and team composition. We looked for opportunities to improve our training program. We identified four key changes that we implemented and surveyed our learners to assess their responses to these changes. All of our key changes were well received by learners. Learning is more than just the content being taught; it is situated in a context where the learning happens [2]. Our changes primarily altered the context under which the technical skills were taught with minimal changes to the informational content, though the content was standardized more as well. Our survey results from our learners support that we made a positive change in our context that the learners found helpful.

The most highly rated change was our instructors acting more as coaches. Coaching is still a work in progress as instructors often revert to instructing and lecturing. Despite this, the change to our emphasis on coaching from instructing was the most important change per the learners’ rankings. The coaching goes hand in hand with the online educational pre-course work and the videos as refreshers. This allows the instructor coaches to feel confident that the information has been shared and the emphasis can be on practicing and mastering the technical skill that the learner is attempting. Future work will need to work on coaching our coaches to help them stay in a coaching mindset and not fall back on old lecture habits. The change to a coaching model, however, has set the learning culture and expectation in our course that allows instructors and learners to give and receive feedback [12]. Our survey results support that learners appreciate this as others have appreciated it as well [13].

The videos were made using principles found in the cognitive theory of multimedia learning. Signaling uses text and symbols on the screen to emphasize key points. Segmenting breaks up complex learning concepts into shorter, focused videos. Weeding uses only the most important points in the video while other less important concepts are not included [6-8]. Our videos use text to emphasize key points, are short and focused, and emphasize the most important points. While our learners may not have known of these concepts in video making exactly, they were able to rank our videos using anchor descriptions on a sliding scale in our survey. Their scores show a very positive response to the quality and educational content of the videos. These well-designed and well-executed videos have resulted in extremely valuable tools for imparting ‘gold standard’ examples of essential NRP concepts and skills. Moreover, we have made them accessible to anyone on YouTube (Playlist: https://youtube.com/playlist?list=PLT3P66D18bGI_BVQNtq8AnVNwAxxlDZPm). Whether incorporated into the in-class learning or used in a flipped classroom model, they may enhance the education of learners in the NRP course.

As a limitation, this study is Tier 1 research and thus not tied to educational retention or patient-level outcomes. Though it is Tier 1, our changes were informed by educational theories and best practices. Also, this study was done as a post-intervention study and there was no pre-intervention data on learning retention to compare with our post-intervention group. Given the access to the videos on YouTube, however, our learners are able to watch them at any time for a refresher and this would almost certainly improve knowledge retention if used. Technical skill performance depends much more on practice and achieving and maintaining mastery. These changes in a course taken once every other year are not likely to dramatically improve later performance. But ongoing coaching and coaching roles in clinical situations have been shown to improve performance such as in positive pressure ventilation and chest compressions [14,15]. Given the learners' improved satisfaction with coaching instructors and its clinical benefits, ongoing technical skill training should focus on using coaching to continue to improve learner skills.

## Conclusions

Our changes have made our NRP class experience better. We altered the context of our learners to better fit the clinical situations we were training them for, and our learners rated these changes highly. Having our instructors adopt a coaching mindset that offered individualized feedback was rated the highest individual change. Instructors should adopt a coaching mindset in all training environments where technical skills are being taught as this has proven benefits to skills education and is highly valued by learners. Video-based content instruction standardized the material presented and the videos were considered professional and extremely helpful to our learners. Wherever NRP skills are being taught, these videos can be accessed and utilized for training. As new changes come to the educational content like the 8th edition, we have been able to retain the context of our learning environment in our in-person portion, and believe they have helped our learners.

## Appendices

Appendix 1: Survey

NRP Skills  Education Change

1. Was this your first time taking the NRP course at TCH in the simulation center? □Yes □No 

Reflecting on your most recent NRP class, please answer all the following questions about the changes to the way the clinical skills portion was taught. In the past after introductions people went around to various skill stations in random order with 2 or 3 other people that may or may not have  been on your final team for the team simulations. Each station had an instructor that usually spent several minutes  teaching  you about the skill and then you practiced and demonstrated the skill. The new format now is after introductions everyone is divided into your team for the remainder of the class. Teams work together to practice the skills needed for resuscitation in the order they appear on the NRP algorithm. The skills are divided into 3 sessions with each session introduced by a video demonstrating and teaching the required knowledge content for the skill session followed by practicing with your instructor coaches. We would like to know more about how this change  has been perceived.

2. On the whole, compared to the previous way the skills were taught, the CURRENT WAY with video review and education of the skills taught in the order they would occur in the NRP algorithm followed by teams performing skills is □Much Worse □Worse □Similar/ Neither better or worse □Better □Much Better

3. Which components of the CURRENT system for skills education did you find to be WORSE than the previous way (CHECK ALL that apply, Multiple answers allowed).

     □Team assignment from the start, staying with the same team for the whole day □Video education and review □Performing skills in the expected order of the NRP algorithm □Concept of instructors as coaches rather than teachers □None of these components were worse

4. Which components of the CURRENT system for skills education did you find to be BETTER than the previous way (CHECK ALL that apply, Multiple answers allowed).

     □Team assignment from the start, staying with the same team for the whole day □Video education and review □Performing skills in the expected order of the NRP algorithm □Concept of instructors as coaches rather than teachers □None of these components were worse

5. Specifically thinking of the videos that introduced each skill session, how would you rate the PRODUCTION QUALITY of the videos? How WELL MADE were they from a VIDEO PRODUCTION standpoint? (Slide the box along the scale continuum left or right to best approximate your opinion)

amateur                                                                            average                                                                     professional

│---------------------------------------------------------------------------------│---------------------------------------------------------------------------------│ (Place a mark on the scale above)

6. Specifically thinking of the videos that introduced each skill session, how would you rate the EDUCATIONAL CONTENT of the videos? To what extent did the videos contain the INFORMATION you needed to perform the skills being taught? (Slide the box along the scale continuum left or right to best approximate your opinion)

no useful information                                     somewhat useful information                        extremely useful information

│---------------------------------------------------------------------------------│---------------------------------------------------------------------------------│ (Place a mark on the scale above)

7. Specifically thinking of the videos that introduced each skill session, how was the PRESENTATION of the information conveyed? To what degree was the content in the video PRESENTED in a CLEAR and EFFECTIVE manner? (Slide the box along the scale continuum left or right to best approximate your opinion)

novice presentation                                             competent presentation                                         expert presentation

│---------------------------------------------------------------------------------│---------------------------------------------------------------------------------│ (Place a mark on the scale above)

8. Specifically thinking of the videos that introduced each skill session, how HELPFUL were the videos in UNDERSTANDING the content for each skill session? To what extent did the videos HELP you UNDERSTAND the skills being taught? (Slide the box along the scale continuum left or right to best approximate your opinion)

not at all helpful                                                       somewhat helpful                                                     extremely helpful

│---------------------------------------------------------------------------------│---------------------------------------------------------------------------------│ (Place a mark on the scale above)

9. OVERALL, my learning and education experience at my LAST CLASS of NRP compared to previous times I took NRP was □Much Worse □Worse □Similar/ Neither better or worse □Better □Much Better

10. Please use this space to add any additional feedback about how we are doing at teaching NRP. What is going well? What can we improve?
